# Metagenomic dataset on lichen *Dirinaria* sp. from the Great Rann of Kutch and tropical moist deciduous Dang forest of Gujarat

**DOI:** 10.1016/j.dib.2020.105504

**Published:** 2020-04-14

**Authors:** Apurvasinh Puvar, Chandrashekar Mootapally, Chaitanya Joshi, Madhvi Joshi

**Affiliations:** a Gujarat Biotechnology Research Centre, Gandhinagar 382011, Gujarat, India; b Hemchandracharya North Gujarat University, Patan, Gujarat, India

**Keywords:** Lichen, Metagenome, NGS, Lichen genomics

## Abstract

This paper describes the additional data to our research article “Bacterial line of defense in Dirinaria lichen from two different ecosystems: First genomic insights of its mycobiont *Diriniria* sp. GBRC AP01” by Puvar et al. [1]. In this manuscript we are presenting the data obtained during the annotation of the genome enriched from metagenomic data from the lichen samples.

Specifications table

**Table:** 

Subject	Applied microbiology and biotechnology
Specific subject area	Metagenomics
Type of data	Table Graph SRA files
How data were acquired	Shotgun sequencing of metagenomes using Ion Proton with 200 bp library chemistry
Data format	Raw Analyzed
Parameters for data collection	*Dirinaria* sp. collected from two varied geographical coordinates (the Great Rann of Kutch: arid, white salt desert; the Dang: tropical moist deciduous forest) of Gujarat. The samples were collected during post-monsoon and further processed with the similar conditions to avoid handling bias in metagenome samples.
Description of data collection	Collected whole lichen thallus along with substrate (rock) from the field and herbariums were prepared for identification at NBRI, Lucknow, Uttar Pradesh, India. Upon collection, the sample for metagenomic DNA isolation were stored in preservative for the purpose.
Data source location	Institution: Gujarat Biotechnology Research center (GBRC) City/Town/Region: Gandhinagar-382011, Gujarat, Country: India Latitude and longitude (and GPS coordinates) for collected samples/data: 23.936806, 69.814500; 20.737083, 73.492278
Data accessibility	Repository name: Metagenomic raw reads submitted to NCBI, the BioProject number is PRJNA526834 Metagenomic data identification number: SRR8731860-61 Direct URL to metagenomic data: https://www.ncbi.nlm.nih.gov/bioproject/526834 MG-RAST: publically available with MG-RAST ID- 87886 (https://www.mg-rast.org/linkin.cgi?project=mgp87886) Skimmed mycobiont Diriniria sp. GBRC AP01 submitted to NCBI, the accession number is SZQE00000000 Mendeley Data: link http://dx.doi.org/10.17632/dfm22pn63b.2#file-8d344350-1cd3–4188–97a5-555d187de100 [2]
Related research article	Puvar, A. C., Nathani, N. M., Shaikh, I., Bhatt, A. D., Bhargava, P., Joshi, C. G., & Joshi, M. N. (2020). Bacterial line of defense in Dirinaria lichen from two different ecosystems: First genomic insights of its mycobiont Dirinaria sp. GBRC AP01. Microbiological Research, 233, 126407. DOI:https://doi.org/10.1016/j.micres.2019.126407

## Value of the data

• The lichen is complex ecosystem having numerous species with majorly fungi and algae or cyanobacteria as symbionts. The Whole shotgun sequence data of lichen from two different eco geographic region revels their diversity, ecosystem and functional attributes in the ecosystem.
• The information supports to the claims by Puvar et al. [1] for KEGG pathway, COG and SEED classifications of the lichen metagenomes.
• Metagenomic data of *Dirinaria* lichen reveals the association of bacteria and mycobiont symbiosis along with associated diversity in response to their natural habitat.
• Functional attributes observed in the lichen metagenomic data will give insight for further elucidation of microbiome role in local biogeochemical cycling.
• The skimmed mycobiont data from lichen metagenome enhances the fungal reference genome database for *Dirinaria* genus.

## 1 Data description

**Fig. 1:**
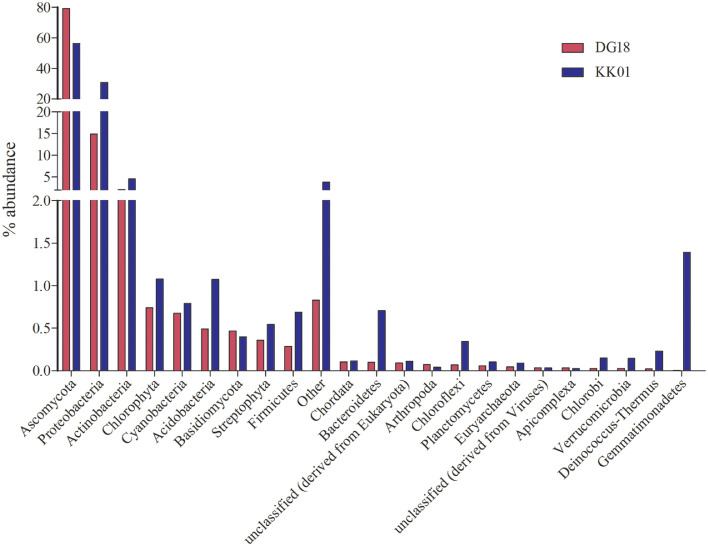
describes the comparative taxonomic profile (top 20 at phylum level) of lichen metagenomes.

**Fig. 2:**
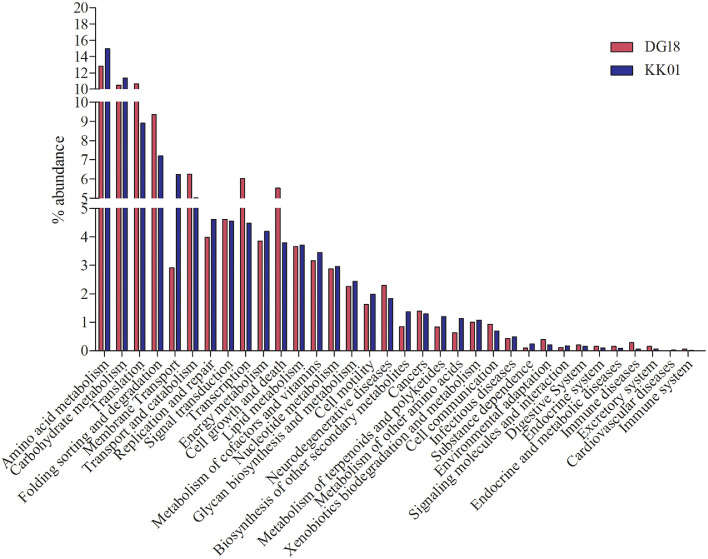
shows the percent difference of top 20 KO (at level2) from both the metagenomes.

**Fig. 3:**
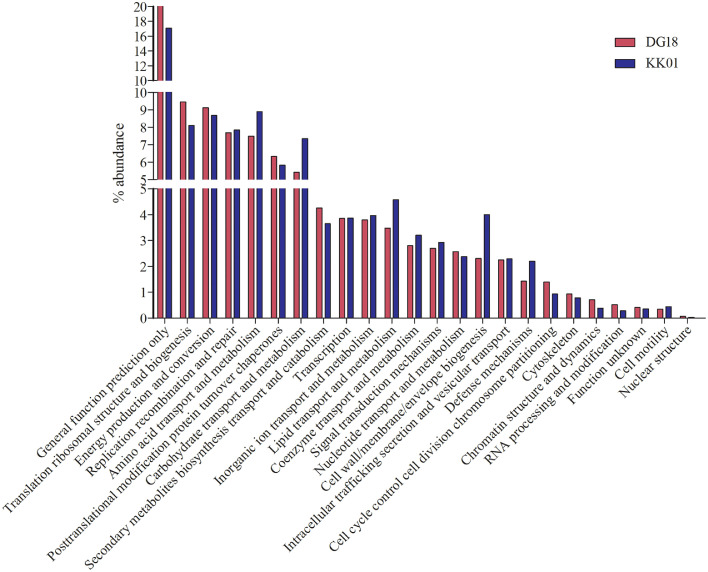
demonstrates variation of COG (top20) at level2 among metagenomes.

**Fig. 4:**
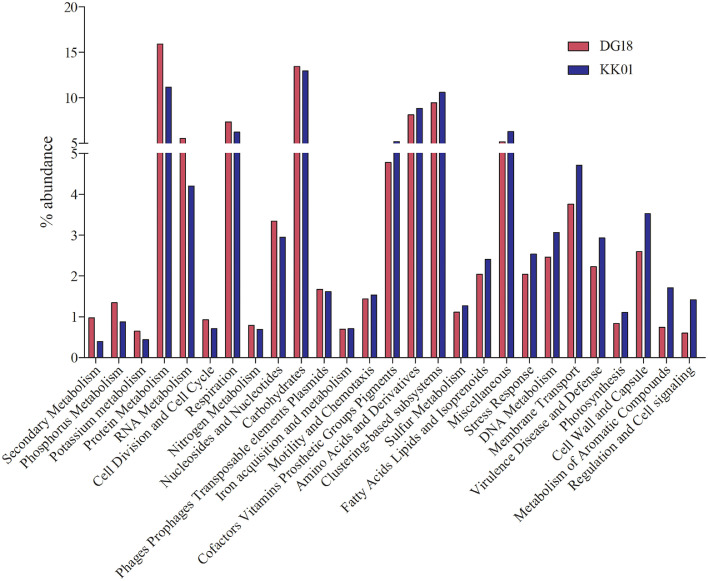
shows top20 abundance difference at level1 of SEED hits in metagenomes.

Metagenomic assemblies from raw data generated in [1] are described in Table 1. which depicts the assembly statistics of the lichen metagenomes. Draft genome was reconstructed using combined approach and basic assembly statistics are described in Table 2. which summarizes the skimmed mycobiont *Diriniria* sp. GBRC AP01. Both metagenomic data were analyzed for taxonomic distribution and their top 20 taxa represented as bar chart in Fig. 1. Similarly, both metagenome assemblies were mapped against KEGG, COG and SEED databases and their relative percent abundance is described in Fig. 2, Fig. 3, Fig. 4 respectively.

**Table 1:** Metegenomic assemblies of data.

Sampling site	Lab ID	Total number of reads (Million)	Total contig number	Total length (Kbp)	Largest contig (Kbp)	N50 (Kbp)
Dang	DG18	5.3	21,532	32,753.60	21.6	2.9
Kutch	KK01	6.3	45,952	39,719.95	18.0	1.3

**Table 2:** Assembly statistics of reconstructed draft genome of *Dirinaria* sp.

Size (Mbp)	Scaffolds (Nos)	N50 (Kbp)	Longest Contig (Kbp)	Genome Completeness
31.67	8624	4.51	27.91	70.3%

## 2 Experimental design, materials and methods

### 2.1 Sampling

*Dirinaria* sp. from two different geographic regions from Gujarat were collected along with their substratum. (a) DG18 was collected from the Dangs district located in southern part of Gujarat covered with semi to dense moist forest. (b) KK01 was collected from Kutch district located western part of the state situated in white salt desert and thorny forest. Both samples were collected during post monsoon seasons along with their substratum i.e. rock.

### 2.2 DNA extraction

Metagenomic DNA was isolated using combined protocol optimized using Qiagen Powersoil kit and Qiagen Plant Mini kit. DNA from each sample (lichen whole thallus) was extracted multiple time to achieve sufficient quantity to perform whole shotgun sequencing i.e. 1 µg DNA in 100 µl volume.

### 2.3 Metagenomic sequencing

About 1 µg of total metagenomic DNA from both samples was used to prepare libraries of 200 bp fragment length using Ion Xpress™ Plus Fragment Library kit and were sequenced using Ion Proton system using Ion PI v2 chip kit [1].

### 2.4 Data analysis

Metagenome assembly was performed using Megahit [3] with the default parameters except minimum contig length as 200 base pairs and the assembly statistics are described in the Table 1.

Both assemblies of *Dirinaria* sp, were submitted to MG-RAST server for taxonomic and functional annotations. The taxonomic (Fig. 1) and functional (Fig. 2–4) annotations of both metagenomes were derived using MG-RAST [4], [5], [6], [7] with default parameters. The mycobiont *Diriniria* sp. GBRC AP01 was skimmed using modified sequence dependent binning approach as described by Albertsen et al., 2013 [8] and genome completeness (Table 2) was assessed using BUSCOs [9].

## Funding Information

This research did not receive any specific grant from funding agencies in the public, commercial, or not-for-profit sectors.
